# Adaptation and psychometric evaluation of a scale to measure oral pre‐exposure prophylaxis‐related stigma among key and vulnerable populations in Kenya

**DOI:** 10.1002/jia2.25929

**Published:** 2022-07-12

**Authors:** Kaitlyn Atkins, Lena Kan, Abednego Musau, Jason Reed, Daniel Were, Diwakar Mohan

**Affiliations:** ^1^ Department of International Health Johns Hopkins Bloomberg School of Public Health Baltimore Maryland USA; ^2^ Jhpiego Kenya Nairobi Kenya; ^3^ Jhpiego Corporation Baltimore Maryland USA

**Keywords:** HIV care continuum, HIV prevention, key and vulnerable populations, PrEP, stigma

## Abstract

**Introduction:**

As oral pre‐exposure prophylaxis (PrEP) services scale up throughout sub‐Saharan Africa (SSA), clients continue to face challenges with sustained PrEP use. PrEP‐related stigma has been shown to influence engagement throughout the HIV PrEP care continuum throughout SSA. Validated quantitative measures of PrEP‐related stigma in SSA are of critical importance to better understand its impacts at each stage of the HIV PrEP care continuum. This study aimed to psychometrically evaluate a PrEP‐related stigma scale for use among key and vulnerable populations in the context of a Kenya national PrEP programme.

**Methods:**

As part of a larger prospective cohort study nested within Kenya's *Jilinde* programme, this study used baseline data collected from 1135 participants between September 2018 and April 2020. We used exploratory factor analysis to evaluate the factor structure of a PrEP‐related stigma scale. We also assessed convergent construct validity of the PrEP‐Related Stigma Scale by testing for expected correlations with depression and uptake of HIV services. Finally, we examined the relationship between PrEP‐related stigma and key demographic, psychosocial and behavioural characteristics.

**Results:**

We identified four dimensions of PrEP‐related stigma: (1) interpersonal stigma, (2) PrEP norms, (3) negative self‐image and (4) disclosure concerns. The scale demonstrated strong internal consistency (α = 0.84), was positively correlated with depressive symptoms and negatively correlated with uptake of HIV services. Multivariable regression analysis demonstrated associations between PrEP‐related stigma and sex worker identity.

**Conclusions:**

The adapted and validated PrEP‐Related Stigma Scale can enable programmes to quantify how PrEP‐related stigma and its dimensions may differentially impact outcomes on the HIV PrEP care continuum, evaluate stigma interventions and tailor programmes accordingly. Opportunities exist to validate the scale in other populations and explore further dimensions of PrEP‐related stigma.

## 1 INTRODUCTION

Since the World Health Organization's 2015 recommendation of pre‐exposure prophylaxis (PrEP) as an HIV prevention option for individuals at risk of HIV infection [1], countries throughout sub‐Saharan Africa (SSA) have rapidly scaled oral PrEP service delivery [2]. While substantial progress has been made in oral PrEP awareness and initiation, research has shown that clients face individual, social and structural challenges with later phases of the HIV PrEP care continuum [3], including sustained PrEP use. One such challenge is PrEP‐related stigma, which has been identified as a barrier for potential and current PrEP users throughout SSA [4, 5, 6, 7]. Several studies have noted the important influence of stigma on decisions around PrEP disclosure and concealment [8, 9, 10], which has implications for support with and consistent use of PrEP.

PrEP‐related stigma refers to stigma associated with the use of PrEP. In the literature, it has been linked to HIV stigma, given PrEP's association with HIV and common conflation of PrEP with HIV treatment. PrEP‐related stigma also encompasses stigma related to sexual norms and behaviour and other perceived risks, rooted in cultural norms [11, 12]. The relationships between PrEP‐related and sexual stigmas are particularly important among key and vulnerable populations (KVP), who have been the target of many PrEP programmes in SSA. For individuals using or considering PrEP, stigma experiences can include enacted stigma (stigmatizing behaviours from others because of PrEP use, including gossip or social exclusion), internalized stigma (shame or negative self‐image related to PrEP use or consideration, based on societal beliefs about PrEP), perceived stigma (perceptions about how PrEP users are treated by others) or anticipated stigma (expectations that others will treat them differently because of PrEP) [13].

Despite emerging qualitative literature around PrEP‐related stigma and its impacts [14, 15, 16, 17, 18, 19, 20], quantitative assessment of PrEP‐related stigma remains sparse. This is particularly true in SSA, where PrEP implementation continues to scale up among diverse populations. Quantitative measures of PrEP‐related stigma are critical to understand and intervene upon its impacts at each stage of the PrEP care continuum, and to evaluate PrEP‐related stigma reduction programmes. This has implications for the broader HIV care continuum, given the potential for PrEP services to improve rates of HIV screening and diagnosis [21]. However, where PrEP‐related stigma scales have been developed, they have only been validated among United States (U.S.) populations [17, 22, 23, 24, 25]. Indeed, we identified only one study from SSA, which used a dedicated scale to measure PrEP‐related stigma, which was adapted from scales developed for U.S. populations [26]. While this scale was found to be internally consistent among the study population, the authors did not report on its validity or other psychometric properties. To fill this critical measurement gap and improve understandings of PrEP‐related stigma, we adapted and psychometrically evaluated a PrEP‐related stigma scale for use among KVP in the context of a nationally scaled PrEP programme in Kenya.

## 2 METHODS

### 2.1 Study setting and sample

This study uses baseline survey data from a prospective cohort study within Kenya's *Jilinde* programme, which has been supporting oral PrEP services in 10 Kenyan counties since 2016 [27]. Through partnership with the Government of Kenya, *Jilinde* supported oral PrEP services for individuals most vulnerable to HIV infections, with a focus on men who have sex with men (MSM), female sex workers (FSW) and adolescent girls and young women (AGYW).

Participants were recruited into the study when accessing oral PrEP services (prescribing, counselling, risk assessment or eligibility determination) at a *Jilinde*‐supported site. After the first clinical visit, PrEP providers briefly introduced the study to potential participants; those interested were referred to trained on‐site data collectors for confirmation of eligibility and consent. Eligible clients had to be MSM, AGYW or FSW who initiated PrEP or were eligible to receive PrEP from a PrEP site. We excluded individuals who were ineligible for PrEP or were unwilling to participate. We did not exclude participants who declined PrEP.

### 2.2 Data collection

Baseline data were collected between September 2018 and April 2020. Surveys were administered on the day of enrolment using an interviewer‐administered questionnaire. Data collectors were experienced, trained quantitative interviewers with degrees or diplomas in health or social sciences. The questionnaire was administered in English, Kiswahili and Dholuo, based on the preferred language of each participant.

We collected data in REDCap using tablets, and uploaded data into a secure REDCap database for analysis [28].

### 2.3 Measures

The baseline questionnaire assessed socio‐demographic characteristics, prior PrEP use, recent sexual behaviour, uptake of HIV‐related services, HIV risk perception, depressive symptoms and perceived PrEP‐related stigma. We also asked participants whether they self‐identified as MSM, AGYW or sex workers (regardless of gender). We offered the option to self‐identify with multiple groups or with none, and categorized participants based on how they identified rather than on their presumed identity during study recruitment (i.e. if men did not self‐identify as MSM, or if women did not identify as either FSW or AGYW, we did not impose these categories).

#### 2.3.1 PrEP‐related stigma

The PrEP‐Related Stigma Scale included 12 items with 4‐point Likert response categories: strongly disagree, disagree, agree and strongly agree. At the time of study development (2017), no validated measures of PrEP‐related stigma existed. We reviewed the literature and determined Reinius et al.’s 12‐item adaptation of the Berger HIV Stigma Scale (HSS) [18, 29] to be most suitable based on language, content and framing of items. We adapted the items to be framed towards future PrEP use by replacing “because you have HIV” with “because you have thought about using PrEP” or “once you have started PrEP” (Table S1). Through consultation with clinicians and members of the KVP community, we made additional adaptations based on the study context and PrEP implementation experiences. For example, for an item which in the HSS asks about perceptions of people living with HIV as dirty, we replaced “dirty” with “immoral” to reflect PrEP use as a behaviour, rather than a condition.

#### 2.3.2 Sexual behaviour

We asked respondents their number of recent (within the past month) partners, condom use with recent (last month) partners (always/sometimes/never) and HIV status of recent partners. For sex workers, we asked about duration of sex work and whether they ever had condomless sex with clients (yes or no).

#### 2.3.3 Uptake of services

We asked if participants had ever accessed HIV‐related services from an MSM‐, sex worker‐ or AGYW‐serving organization. For those who had, we calculated their total reported number of visits in the last 12 months to measure uptake of HIV‐related services at KVP‐serving organizations; those who had not ever accessed such services were assigned a value of 0 visits in the last 12 months. We also asked whether they had previously been offered PrEP (yes/no).

#### 2.3.4 HIV risk perception

We assessed HIV risk perception using a single question: “To what extent do you feel vulnerable to/at risk for HIV infection from any source?” Response options included high risk, medium risk, low risk or no risk.

#### 2.3.5 Depressive symptoms

We measured depressive symptoms using the Patient Health Questionnaire‐9 (PHQ‐9; Cronbach's alpha = 0.84) [30]. The PHQ‐9 has been validated among adults living with HIV and community members in Kenya [31], and is increasingly used to evaluate psychological distress with vulnerable populations in SSA [32, 33, 34, 35]. We used total PHQ‐9 scores, which ranged from 0 to 27; consistent with previous research in the region [36], we classified individuals with scores of 10 or above as having symptoms suggestive of depression. Respondents who did not respond to all nine items (*n* = 22) were classified as missing.

### 2.4 Statistical analysis

We calculated descriptive statistics for socio‐demographic characteristics, partner HIV status, number of recent partners and primary partner's HIV status, history with PrEP, depression and uptake of HIV services from KVP‐serving organizations.

### 2.5 Evaluation of factor structure

To determine the number of factors to extract during exploratory factor analysis (EFA), we first examined the results of a principal component analysis (PCA) [37]. Given all items had 4‐point Likert response categories, PCA was performed with a polychoric correlation matrix of item responses. Selection of number of factors for EFA was informed by examination of a scree plot, parallel analysis and the number of eigenvalues >1.0.

Based on an omnibus test revealing deviation from normality, we used iterative principal factor estimation for EFA, with oblique rotation to yield a final solution. Items with loadings on a single factor of at least 0.40 and uniqueness below 0.50 were retained.

### 2.6 Assessment of internal consistency and construct validity

Based on the factor structure, we developed four subscales using simple mean scores. Each subscale had three items, with scores ranging from 0 to 3; a score of 3 indicated respondents “strongly agreed” with all items. We also generated a total PrEP‐related stigma score by summing mean subscale scores, with total scores ranging from 0 to 12. As with subscale scores, higher total scores indicated higher levels of PrEP‐related stigma. We used Cronbach's alpha and McDonald's omega to assess internal consistency of the overall scale and subscales. We also evaluated item‐test and inter‐item correlations.

We assessed convergent construct validity of the PrEP‐Related Stigma Scale by testing for expected correlations between PrEP‐related stigma (total score and subscale scores) and depressive symptoms (composite PHQ‐9 score), as well as PrEP‐related stigma and uptake of HIV services (total visits in the last 12 months). We hypothesized that PrEP‐related stigma would have a positive correlation with depressive symptoms and a negative correlation with HIV services uptake.

### 2.7 Factors associated with stigma

To examine how this scale could be used in research settings and to profile who may be more likely to experience PrEP‐related stigma, we used linear regression to estimate associations between the outcome of PrEP‐related stigma and socio‐demographic characteristics hypothesized to be related to stigma. We first examined bivariate, unadjusted associations between total PrEP‐related stigma scores and age, gender, HIV risk perception, partner HIV status, prior offers of PrEP and population group. Among FSW, we also examined associations with years in sex work. We then estimated four adjusted multivariable models: one with the full sample and one each with AGYW, FSW and MSM. Adjusted models included all variables found to be associated with PrEP‐related stigma in unadjusted models based on a cut‐off of *p* <0.25.

All analyses were conducted in Stata Version 15.

### 2.8 Ethical considerations

Ethics approvals for the study were obtained from the Kenya Medical Research Institute Scientific Ethics Review Unit and the Johns Hopkins Bloomberg School of Public Health Institutional Review Board. All data collectors received training on human subject protections and gender and sexual diversity. Study protocols ensured that sensitive questions were only asked when participants had been informed about the questions and were ready to continue. All participants provided written informed consent, with the option to omit names from consent forms or use a thumbprint.

## 3 RESULTS

In total, 1196 individuals were referred by providers and screened for study inclusion. Of 1181 eligible, 46 declined participation. Our final sample included 1135 participants who completed the baseline questionnaire (Table 1). Just over half (56.6%) identified as sex workers (55.2% FSW and 1.4% male sex workers). Less than one‐tenth (9.3%) were MSM, and 26.7% were AGYW; 79 respondents (7.0%) did not identify as MSM, sex workers or AGYW.

**Table 1:** Characteristics of participants at baseline

	Full sample (*n* = 1135)^a^	AGYW (*n* = 303)^c^	FSW (*n* = 626)^c^	MSM (*n* = 105)^c^
Age (median [IQR])	24 [20–29]	20 [18–22]	27 [22–32]	23 [21–25]
Self‐reported gender identity				
Man	104 (9.2%)	0 (0.0%)	0 (0.0%)	81 (77.9%)
Woman	999 (88.6%)	302 (100.0%)	626 (100.0%)	0 (0%)
Other^b^	25 (2.2%)	0 (0.0%)	0 (0.0%)	23 (22.1%)
Geographic region				
Nairobi (Nairobi, Machakos and Kiambu Counties)	292 (25.8%)	6 (2.0%)	179 (28.6%)	84 (80.0%)
Lake (Kisumu, Migori and Kisii Counties)	434 (38.3%)	289 (95.4%)	108 (17.3%)	17 (16.2%)
Coast (Mombasa, Kilifi and Kwale Counties)	407 (35.9%)	8 (2.6%)	338 (54.1%)	4 (3.8%)
Education level				
Less than primary	118 (10.4%)	32 (10.6%)	66 (10.5%)	1 (1.0%)
Primary	554 (48.9%)	172 (56.8%)	327 (52.2%)	17 (16.2%)
Secondary	353 (31.2%)	74 (24.4%)	188 (30.0%)	60 (57.1%)
Tertiary	107 (9.5%)	25 (8.3%)	45 (7.2%)	27 (25.7%)
Employment status				
Unemployed/student	492 (43.5%)	239 (78.9%)	161 (25.8%)	59 (56.7%)
Self‐employed	407 (36.0%)	36 (11.9%)	314 (50.2%)	18 (17.3%)
Regularly employed, part‐time	80 (7.1%)	9 (3.0%)	50 (8.0%)	12 (11.5%)
Regularly employed, full‐time	47 (4.2%)	12 (4.0%)	21 (3.4%)	7 (6.7%)
Seasonally employed	104 (9.2%)	7 (2.3%)	79 (12.6%)	8 (7.7%)
Gross monthly income, USD (median [IQR])	44 [1–131]	0 [0–1]	79 [35–153]	52 [1–131]
Marital status				
Unmarried	917 (81.1%)	191 (63.2%)	562 (89.8%)	93 (88.6%)
Married	156 (13.8%)	105 (34.8%)	28 (4.5%)	7 (6.7%)
Domestic partnership	57 (5.0%)	5 (1.7%)	36 (5.8%)	5 (4.8%)
Number of recent sex partners				
1	285 (26.6%)	197 (71.6%)	27 (4.4%)	26 (27%)
2	198 (18.5%)	55 (20.0%)	80 (13.0%)	28 (29%)
3 or more	587 (54.9%)	23 (8.4%)	509 (82.6%)	42 (44%)
Partner HIV status^d^				
Living with HIV	12 (5.7%)	4 (3.7%)	5 (8.0%)	0 (0%)
Not living with HIV	118 (55.7%)	64 (58.7%)	35 (55.0%)	11 (92.0%)
Unknown HIV status	82 (38.7%)	41 (37.6%)	24 (38.0%)	1 (8.0%)
Ever offered PrEP before				
Yes	75 (6.6%)	27 (8.9%)	35 (5.6%)	12 (11.4%)
No	1056 (93.4%)	275 (91.1%)	590 (94.4%)	93 (88.6%)
HIV risk perception				
No risk	50 (4.5%)	21 (7.1%)	17 (2.7%)	6 (5.7%)
Low risk	143 (12.8%)	52 (17.7%)	50 (8.0%)	26 (24.8%)
Medium risk	416 (37.3%)	143 (48.6%)	214 (34.3%)	45 (42.9%)
High risk	507 (45.4%)	78 (26.5%)	342 (54.9%)	28 (26.7%)
Depressive symptoms				
Not suggestive of depression	868 (78.0%)	264 (88.9%)	442 (71.2%)	89 (84.8%)
Suggestive of depression	245 (22.0%)	33 (11.1%)	179 (28.8%)	16 (15.2%)
Uptake of HIV services, last 12 months				
No visits	446 (43.2%)	74 (24.7%)	311 (50.1%)	51 (48.6%)
1–2 visits	315 (30.5%)	113 (37.7%)	166 (26.7%)	37 (35.2%)
3–6 visits	242 (23.4%)	105 (35.0%)	127 (20.5%)	13 (12.4%)
7 or more visits	29 (2.8%)	8 (2.7%)	17 (2.7%)	4 (3.8%)

^a^Missing values not shown.

^b^Includes transgender men (*n* = 4), transgender women (*n* = 18), intersex individuals (*n* = 2) and those with other, unknown or unreported gender identities (*n* = 5).

^c^Not mutually exclusive. Respondents could select all group identities that applied, though MSM and male sex worker categories were only presented to self‐identified men and FSW/AGYW categories to self‐identified women. No men reported multiple categories; 13 women respondents reported identifying as both AGYW and FSW. 16 men identified as male sex workers. 79 individuals reported not identifying as any risk group.

^d^Among those reporting being married or in domestic partnerships (*n* = 213).

Abbreviations: AGYW, adolescent girls and young women; FSW, female sex workers; IQR, interquartile range; MSM, men who have sex with men; PrEP, pre‐exposure prophylaxis; USD, United States Dollar.

The median age was 24 years (interquartile range [IQR]: 20–29); FSW were slightly older than others (median age 27, IQR 22–32). Most participants (88.6%) self‐identified as women, and over half (59.3%) had completed primary school or less. The majority (81.2%) were unmarried, and 54.9% reported three or more recent (last month) sexual partners. A greater proportion of AGYW were married (34.8%) than FSW (4.5%) or MSM (6.7%), and most AGYW (71.6%) had only one recent partner. Of those with primary partners, 38.7% reported not knowing their partner's HIV status. Most respondents (93.4%) had never been offered PrEP before the initial visit. Prior offers of PrEP were most common among MSM (11.4%).

In terms of HIV risk perception, 4.4% of respondents felt that they were not vulnerable to HIV infection, while 44.9% felt that they were at high risk. The majority of FSW (54.9%) reported feeling at high risk, compared with smaller percentages of AGYW (26.5%) and MSM (26.7%). Reports of visiting sex worker‐, MSM‐ or AGYW‐serving organizations for HIV services varied, with 43.2% of respondents saying they had not visited these sites in the last 12 months, 30.5% reporting one or two visits and 2.8% reporting seven or more visits. FSW (50.1%) and MSM (48.6%) more commonly reported not taking up HIV services than AGYW (24.7%). Finally, symptoms suggestive of depression were identified in 22.0% of respondents, with over one‐quarter (28.8%) of FSW reporting depressive symptoms.

### 3.1 PrEP‐Related Stigma Scale factor structure

PCA of the 12 items produced four eigenvalues over one (range: 1.1–5.2) that together explained 80% of the variance. Examination of the scree plot and parallel analysis similarly favoured a four‐factor model. Results from this factor analysis with oblique rotation are provided in Table 2. Factor loadings ranged from 0.70 to 0.94, with all items loading strongly onto at least one factor (loadings >0.40) and no items having uniqueness <0.50. As such, no items were dropped from the analysis.

**Table 2:** PrEP‐Related Stigma Scale item means, factor loadings and factor correlations (*n* = 1135)

	Factor loadings^b^				
Items^a^	Factor 1: Inter‐personal stigma (anticipated)	Factor 2: PrEP norms (perceived)	Factor 3: Negative self‐image (internalized)	Factor 4: Disclosure concerns (anticipated)	Uniqueness
1. Are you afraid people you care about will stop calling after learning you have started or thought of using PrEP?	**0.84**	0.03	–0.01	–0.01	0.28
2. Are you afraid of losing friends if you tell them you have started or thought of using PrEP?	**0.94**	–0.03	–0.02	0.00	0.16
3. Some people might avoid touching you once they know you have started or thought of using PrEP.	**0.72**	0.04	0.09	0.01	0.37
4. You would work hard to keep your use of PrEP a secret.	0.07	–0.01	0.01	**0.83**	0.26
5. Telling someone you have thought of using PrEP is risky.	0.07	–0.07	0.02	**0.74**	0.45
6. You will be very careful who you tell that you have thought of using PrEP.	–0.11	0.08	–0.03	**0.85**	0.28
7. Most people you know believe a person who takes PrEP is immoral.	–0.01	**0.73**	–0.07	0.07	0.47
8. People you know who take PrEP are treated like outcasts.	–0.01	**0.87**	0.05	–0.02	0.23
9. Most people you know are uncomfortable around someone who takes PrEP.	0.08	**0.70**	0.11	0.01	0.35
10. You feel guilty because you have thought of using PrEP.	–0.02	0.05	**0.85**	0.00	0.25
11. People's attitudes about using PrEP make you feel worse about yourself.	0.04	0.07	**0.81**	0.00	0.25
12. You feel you are not as good a person as others because you have thought of using PrEP.	0.00	–0.05	**0.92**	0.00	0.19
	Internal consistency reliability coefficients				
Cronbach's alpha (α)	α = 0.83	α = 0.78	α = 0.84	α = 0.80	
McDonald's omega (ω)	ω = 0.84	ω = 0.79	ω = 0.84	ω = 0.80	

	Factor correlations			
	Factor 1	Factor 2	Factor 3	Factor 4
Factor 1: Interpersonal stigma	1			
Factor 2: PrEP norms	0.49	1		
Factor 3: Negative self‐image	0.48	0.51	1	
Factor 4: Disclosure concerns	0.38	0.42	0.15	1

Abbreviation: PrEP, pre‐exposure prophylaxis.

^a^All item responses were on a 4‐point range from “strongly disagree” to “strongly agree.” These items have been shortened; their exact wording is listed in the Supplementary File.

^b^The highest factor loading for each item is bolded.

We identified four factors (Table 2), which were named interpersonal stigma (factor 1), PrEP norms (factor 2), negative self‐image (factor 3) and disclosure concerns (factor 4). In Table 2, we have indicated the type of stigma measured by each factor (internalized, perceived and anticipated).

Factor correlations were moderately positive, with the highest correlations observed between factors 1 and 2 (interpersonal stigma and PrEP norms; *r* = 0.49) and factors 2 and 3 (PrEP norms and negative self‐image; *r* = 0.51). Factors 3 and 4 (negative self‐image and disclosure concerns) had the weakest correlation (*r* = 0.15).

### 3.2 Internal consistency and construct validation

For the internal consistency analysis of the full scale, the Cronbach's alpha and McDonald's omega were both 0.84. Cronbach's alpha (α) values for subscales ranged from 0.78 to 0.84 (interpersonal stigma α = 0.83; PrEP norms α = 0.78; negative self‐image α = 0.84; and disclosure concerns α = 0.80). McDonald's omega (ω) values ranged from 0.79 to 0.84 (interpersonal stigma ω = 0.84; PrEP norms ω = 0.79; negative self‐image ω = 0.84; and disclosure concerns ω = 0.80). The average inter‐item correlation was 0.31, and all item‐test correlations were >0.50.

Correlations between PrEP‐related stigma scores with other constructs are presented in Table 3. Overall, PrEP‐related stigma demonstrated a significantly small but positive correlation with depression, and small but negative correlation with uptake of HIV services. Subscales were significantly positively correlated with depression; only the factor 2 (PrEP norms) and factor 3 (negative self‐image) subscales were significantly correlated with HIV services uptake.

**Table 3:** Correlation matrix of PrEP‐related stigma, depression and engagement with PrEP services

	PrEP‐related stigma: total score	Factor 1: Inter‐personal stigma	Factor 2: PrEP norms	Factor 3: Negative self‐image	Factor 4: Disclosure concerns	PHQ‐9 total score	HIV services uptake
PrEP‐related stigma (total score)	1.0						
Factor 1: Inter‐personal stigma	0.73^***^	1.0					
Factor 2: PrEP norms	0.76^***^	0.41^***^	1.0				
Factor 3: Negative self‐image	0.64^***^	0.39^***^	0.40^***^	1.0			
Factor 4: Disclosure concerns	0.69^***^	0.31^***^	0.35^***^	0.15^***^	1.0		
Depression (PHQ‐9 total score)	0.20^***^	0.10^*^	0.20^***^	0.09^**^	0.15^***^	1.0	
HIV services uptake (visits last 12 months)	–0.07^*^	–0.02	–0.08^*^	–0.07^*^	–0.03	0.07^*^	1.0

^*^ *p* <0.05.

^**^ *p* <0.01.

^***^ *p* <0.001.

### 3.3 PrEP‐related stigma prevalence and associated factors

Out of 12 possible points, the mean overall PrEP‐related stigma score was 4.3 (SD 1.9). On subscales, scores were highest for the disclosure concerns subscale (mean 1.8, SD 0.8) and lowest for negative self‐image (mean 0.5, SD 0.6). Scores averaged at 0.8 of 3 (SD 0.7) for interpersonal stigma and 1.1 of 3 (SD 0.7) for PrEP norms.

**Figure 1:**
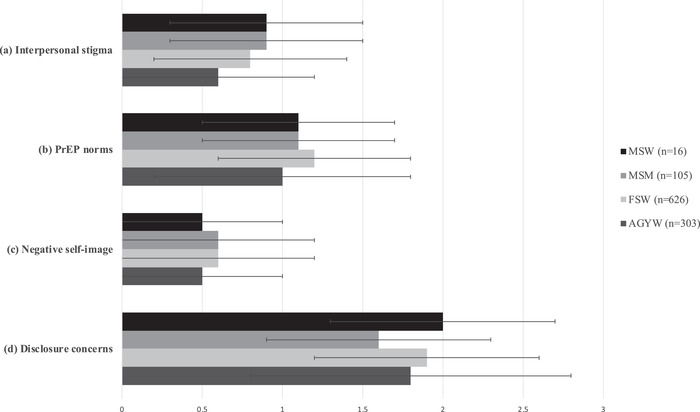
Means and standard deviations for PrEP‐related stigma scores by subgroup (*n* = 1135). (a) Interpersonal stigma subscale; (b) PrEP norms subscale; (c) negative self‐image subscale; and (d) disclosure concerns subscale. Shaded bars represent mean values for each scale per subgroup; error bars represent standard deviations from the mean. In each group (a–d), black (top) bars represent male sex workers (MSW, *n* = 16); medium grey (second from top) bars represent men who have sex with men (MSM, *n* = 105); light grey (third from top) bars represent female sex workers (FSW, *n* = 616); and dark grey (bottom) bars represent adolescent girls and young women (AGYW, *n* = 303). Possible scores ranged from 0 to 3 for each subscale (a–d). Abbreviations: PHC‐9, Patient Health Questionnaire‐9; PrEP, pre‐exposure prophylaxis.

In subgroup analyses, overall PrEP‐related stigma scores were higher among MSW (mean 4.6, SD 1.7) and FSW (mean 4.5, SD 1.7) compared to AGYW (mean 3.9, SD 2.2) or MSM (mean 4.3, SD 2.0). On subscales (Figure 1), MSM scored highest on the interpersonal stigma subscale (mean 0.9, SD 0.6), while FSW scored highest on the PrEP norms subscale (mean 1.2, SD 0.6) and MSW highest on the disclosure concerns subscale (mean 2.0, SD 0.7). Across groups, scores were highest on the disclosure concerns subscale and lowest on the negative self‐image subscale.

In unadjusted models (Table 4), PrEP‐related stigma was found to be associated with increased age (β = 0.02, *p* <0.01), medium HIV risk perception (β = 0.62, *p* = 0.03), previous offers of PrEP (β = –0.50, *p* = 0.03) and identifying as a sex worker (β = 0.46, *p* <0.001). In a multivariable model with the full sample (model 1), PrEP‐related stigma was shown to be associated with identifying as a sex worker; adjusting for covariates, sex workers reported higher levels of PrEP‐related stigma than others (β = 0.49, *p* <0.001). In subgroup analyses with AGYW (model 2) and MSM (model 4), no variables were found to be associated with PrEP‐related stigma. In the model with FSW (model 3), we found that FSW who reported low HIV risk perception had higher levels of PrEP‐related stigma, adjusting for covariates (β = 0.97, *p* = 0.04).

**Table 4:** Linear regression of PrEP‐related stigma on key characteristics

	Unadjusted associations		Model 1: Full sample (*n* = 1135)		Model 2: AGYW (*n* = 303)		Model 3: FSW (*n* = 626)		Model 4: MSM (*n* = 105)	
Characteristic	β	SE	β	SE	β	SE	β	SE	β	SE
Age	0.02^**^	0.01	0.13	0.09	0.02	0.04	0.01	0.01	0.03	0.04
Self‐reported gender identity										
Woman	REF	REF	REF	REF	–	–	–	–	–	–
Man	0.14	0.20	0.39	0.21	–	–	–	–	–	–
Other	–0.44	0.39	–0.05	0.39	–	–	–	–	–	–
Perceived HIV risk										
No risk	REF	REF	REF	REF	REF	REF	REF	REF	REF	REF
Low risk	0.48	0.32	0.42	0.31	0.19	0.58	0.97^*^	0.47	–0.89	0.92
Medium risk	0.62^*^	0.29	0.53	0.29	0.63	0.52	0.73	0.42	–0.76	0.88
High risk	0.42	0.28	0.21	0.28	0.70	0.55	0.29	0.42	–0.88	0.91
Partner HIV status^a^										
Living with HIV	REF	REF	–	–	–	–	–	–	–	–
Not living with HIV	–0.59	0.62	–	–	–	–	–	–	–	–
Unknown HIV status	0.14	0.14	–	–	–	–	–	–	–	–
Previously offered PrEP										
No	REF	REF	REF	REF	REF	REF	REF	REF	REF	REF
Yes	–0.50^*^	0.23	–0.51^*^	0.23	–0.67	0.46	–0.74^*^	0.29	0.03	0.64
Identifies as MSM^a^										
No	REF	REF	–	–	–	–	–	–	–	–
Yes	0.40	0.20	–	–	–	–	–	–	–	–
Identifies as sex worker										
No	REF	REF	REF	REF	–	–	–	–	–	–
Yes	0.46^**^	0.12	0.49^**^	0.14	–	–	–	–	–	–
Years in sex work^b^										
Less than 1 year	REF	REF	–	–	–	–	REF	REF	–	–
1–2 years	0.34	0.33	–	–	–	–	0.26	0.33	–	–
3–5 years	0.34	0.33	–	–	–	–	0.31	0.33	–	–
6–9 years	0.57	0.38	–	–	–	–	0.44	0.39	–	–
10+ years	–0.07	0.41	–	–	–	–	–0.27	0.43	–	–

Abbreviations: AGYW, adolescent girls and young women; FSW, female sex workers; MSM, men who have sex with men; PrEP, pre‐exposure prophylaxis.

^a^ Variable excluded from multivariate models based on *p* >0.25 during bivariate analysis.

^b^ Among those reporting sex work.

^*^ *p* <0.05.

^**^ *p* <0.01.

## 4 DISCUSSION

This is among the first studies to adapt and evaluate the psychometric properties of a scale to measure PrEP‐related stigma in SSA. The final scale included 12 items. Factor analysis revealed a four‐factor structure, corresponding to dimensions interpersonal stigma (factor 1), PrEP norms (factor 2), negative self‐image (factor 3) and disclosure concerns (factor 4). The scale demonstrated strong internal consistency and appropriate convergent construct validity.

The four dimensions identified through this analysis align closely with those in the HSS from which our measure was adapted [29]. While this is to be expected given close alignment of the items (S1), our adaptation offers a validated measure to examine these dimensions as they relate to PrEP more specifically. While previously validated measures of PrEP‐related stigma are limited to U.S. contexts, it is worth examining how our scale aligns with these existing measures. Similar to our scale, Siegler et al.’s 13‐item measure of PrEP stigma [24] examined perceived, anticipated and internalized stigma; however, unlike our multidimensional scale, it was found to be unidimensional. Klein and Washington's shortened 11‐item PrEP Stigma Scale [38] identified two high‐performing dimensions of stigma, including a dimension of interpersonal concerns, which aligns with our interpersonal stigma dimension (factor 1). Our PrEP norms dimension aligns with several other measures, which have PrEP user stereotypes and community norms to be key dimensions of PrEP‐related stigma [17, 22, 25]. Others have identified dimensions of PrEP stigma unmeasured in our study but important for future research. For example, Algarin et al.’s multidimensional Community PrEP‐Related Stigma Scale [25] identified a dimension of positive community perceptions of PrEP.

We found that PrEP‐related stigma was associated with sex worker identity, and that PrEP‐related stigma was generally more prevalent among FSW than among MSM or AGYW. This sheds light on the ways in which PrEP‐related stigma experiences may meaningfully vary based on the diverse identities and social positions of PrEP users. In our study, respondents identifying as sex workers (regardless of gender) reported higher levels of anticipated, perceived and internalized PrEP‐related stigma, suggesting PrEP‐related stigma may be different among sex workers than others. While we did not measure sex work stigma directly, this relates to other literature describing intersecting PrEP and sex work stigmas [8, 11, 39]. Indeed, scholars have identified intersections between PrEP‐related stigma and other forms of stigma and discrimination (including racial discrimination, transphobia, sexual stigma and others) in other populations [7, 17, 40, 41]. Further exploration of these intersections in SSA contexts is warranted through the use of parallel measures examining different identities and social positions [42].

Our PrEP‐Related Stigma Scale makes an important contribution to the literature regarding stigma as a determinant of engagement with HIV services. Though much previous research regarding stigma and HIV services has focused on engagement in care and treatment for PLHIV [43, 44], this new scale enables more focused examination of stigma among individuals engaging with or considering an HIV prevention intervention. This valid and reliable instrument enables programmes to consider how PrEP‐related stigma and its dimensions may differentially impact engagement with PrEP services, tailor programmes accordingly and evaluate the impacts of stigma reduction or mitigation programmes. For example, our finding that PrEP‐related stigma was higher among those reporting sex work suggests a need for tailored stigma mitigation interventions among this population. Further, our finding that disclosure concerns were more common than negative self‐image related to PrEP suggests a need for tailored support and safety planning to PrEP users who may conceal their PrEP use [45, 46, 47]. This scale also enables researchers to examine the impacts of PrEP‐related stigma on other HIV care continuum outcomes, such as uptake of HIV testing during PrEP services, PrEP initiation, adherence and continuation or other patterns of PrEP use.

### 4.1 Limitations

There are limitations to this research. First, the generalizability of our sample is limited, as our recruitment protocol which may have resulted in a sample highly motivated about PrEP. Further, we focused on three geographic regions in Kenya and the majority of our respondents were women and had partners who were either not living with HIV or had unknown HIV status. Second, while all participants in our study were new PrEP clients who had received counselling and education about PrEP through *Jilinde*, they may have had differing levels of prior exposure to PrEP, which may have influenced stigma. We were unable to thoroughly assess this prior exposure in our survey. Third, this was a cross‐sectional analysis limited to baseline data, which limits the ability to draw causal inferences from our regression analysis. Longitudinal analysis was outside the scope of this study, but further analyses should examine the impacts of PrEP‐related stigma at later stages of the HIV PrEP care continuum, including PrEP discontinuation and patterns of cycling PrEP use. Fourth, our study involved a close adaptation of an existing measure, which was designed to evaluate broader HIV stigma, rather than PrEP‐related stigma specifically. While previous research has shown that PrEP‐related stigma is highly related to HIV stigma, it is possible that there are additional dimensions of PrEP‐related stigma not assessed with our adapted instrument. For example, previous work has found that PrEP is often associated with negative attitudes towards sexual practices and behaviour, such as sexual activity among AGYW, sex work or same‐sex partnerships [7]. These relationships are context‐dependent and warrant further investigation. Finally, we were not able to assess enacted PrEP‐related stigma (e.g. “Has anyone ever gossiped about you because you use PrEP?”), as this was a baseline assessment among individuals who had not yet started PrEP. This may limit the generalizability of our scale to assess stigma experiences among current PrEP users.

## 5 CONCLUSIONS

The PrEP‐Related Stigma Scale has been shown as an appropriate scale for use among diverse communities in Kenya. This instrument, which was validated and found reliable in our study population, offers researchers a tool for quantifying experiences of anticipated, perceived and internalized PrEP‐related stigma among diverse populations, to better understand its impacts on engagement with the HIV PrEP care continuum. Opportunities exist to validate the scale in other populations and to explore other dimensions of PrEP‐related stigma, including those that may vary based on the unique identities and positions of PrEP users.

## COMPETING INTERESTS

The authors declare no competing interests.

## AUTHORS’ CONTRIBUTIONS

DW, AM and JR conceptualized the study. KA and LK analysed the data, with supervision from DM. KA drafted the manuscript. LK, AM, JR, DW and DM provided critical review and feedback on the draft. All authors have read and approved the final manuscript.

## FUNDING

The Jilinde project received funding from the Bill & Melinda Gates Foundation under award number INV‐007340. KA received support from the National Institute of Mental Health (1F31MH124583). In addition, the project received a donation of PrEP commodities (TDF/FTC) from Gilead Health Sciences.

## DISCLAIMER

The views expressed in this manuscript are those of the authors and do not necessarily represent the opinions of the project donors.

## Supporting information


**Table S1**. Final PrEP Stigma Scale items.

## Data Availability

Jilinde Prospective Cohort Data are available online via prerelease at: https://clinepidb.org/ce/app/workspace/analyses/DS_9c28dda6c7.
