# Supplementary material for: Adaptation and psychometric evaluation of a scale to measure oral pre‐exposure prophylaxis‐related stigma among key and vulnerable populations in Kenya
Source: J Int AIDS Soc. 2022 Jul 12;25(Suppl 1):e25929. doi: 10.1002/jia2.25929 (PMC9274213; doi:10.1002/jia2.25929)
Supplement: Supplementary file 1 — Table S1. Final PrEP Stigma Scale items. [file JIA2-25-e25929-s001.docx]

**Table S1.** Final PrEP Stigma Scale items.

| **PrEP Stigma Scale Item** | **Corresponding Berger HSS Item** |
| --- | --- |
| Are you afraid people you care about will stop calling after learning you have started or thought of using PrEP? | People I care about stopped calling after learning I have HIV. |
| Are you afraid of losing friends if you tell them you have started or thought of using PrEP? | I have lost friends by telling them I have HIV. |
| Some people might avoid touching you once they know you have started or thought of using PrEP. | People avoid touching me if they know I have HIV. |
| You would work hard to keep your use of PrEP a secret. | I work hard to keep my HIV a secret. |
| Telling someone you have started or thought of using PrEP is risky. | Telling someone I have HIV is risky. |
| You will be very careful who you tell that you have started or thought of using PrEP. | I am very careful whom I tell that I have HIV. |
| Most people you know believe a person who takes PrEP is immoral. | Most people believe a person who has HIV is dirty. |
| People you know who take PrEP are treated like outcasts. | People with HIV are treated like outcasts. |
| Most people you know are uncomfortable around someone who takes PrEP. | Most are uncomfortable around someone with HIV. |
| You feel guilty because you have started or thought of using PrEP. | I feel guilty because I have HIV. |
| People’s attitudes about using PrEP make you feel worse about yourself. | People’s attitudes [about HIV] make me feel worse about myself. |
| You feel you are not as good a person as others because you have started or thought of using PrEP. | I feel I’m not as good as others because I have HIV. |
